# Effect of Silicate and Phosphate Solubilizing Rhizobacterium *Enterobacter ludwigii* GAK2 on *Oryza sativa* L. under Cadmium Stress

**DOI:** 10.4014/jmb.1906.06010

**Published:** 2019-10-25

**Authors:** Arjun Adhikari, Ko-Eun Lee, Muhammad Aaqil Khan, Sang-Mo Kang, Bishnu Adhikari, Muhammad Imran, Rahmatullah Jan, Kyung-Min Kim and In-Jung Lee

**Affiliations:** School of Applied Biosciences, Kyungpook National University, Daegu 41566, Republic of Korea

**Keywords:** Cadmium, *Enterobacter ludwigii* GAK2, phosphorus, rice

## Abstract

Silicon and phosphorus are elements that are beneficial for plant growth. Despite the abundant availability of silicate and phosphate in the Earth’s crust, crop nutritional requirements for silicon and phosphorus are normally met through the application of fertilizer. However, fertilizers are one of the major causes of heavy metal pollution. In our study, we aimed to assess silicate and phosphate solubilization by the bacteria *Enterobacter ludwigii* GAK2, in the presence and absence of phosphate [Ca_3_(PO_4_)_2_] or silicate (Mg_2_O_8_Si_3_), to counteract cadmium stress in rice (*Oryza sativa* L). Our results showed that the GAK2-treated rice plants, grown in soil amended with phosphate [Ca_3_(PO_4_)_2_] or silicate (Mg_2_O_8_Si_3_), had significantly reduced cadmium content, and enhanced plant growth promoting characteristics including fresh shoot and root weight, plant height, and chlorophyll content. These plants showed significant downregulation of the cadmium transporter gene, *OsHMA2*, and upregulation of the silicon carrier gene, *OsLsi1*. Moreover, jasmonic acid levels were significantly reduced in the GAK2-inoculated plants, and this was further supported by the downregulation of the jasmonic acid related gene, *OsJAZ1*. These results indicate that *Enterobacter ludwigii* GAK2 can be used as a silicon and phosphorus bio-fertilizer, which solubilizes insoluble silicate and phosphate, and mitigates heavy metal toxicity in crops.

## Introduction

Heavy metals have been widely disseminated for sustainable agriculture and human well-being [1]. They have been excessively exposed in the environment through anthropogenic activities. Although, many of these metals under threshold concentrations serve as vital nutrients for plant growth, when they exceed their threshold limits, they impose toxicity in nature [1]. Heavy metals such as cadmium (Cd) are highly toxic to plants, even at low concentrations [2]. Its threshold value in soil is 1 mg/kg, and it presents ecological risks if levels in soil exceed 10 mg/kg [2, 3]. Furthermore, Cd is ranked as the 7^th^ most highly toxic element [4].

Phosphate fertilizers are considered as the major causative factor for Cd contamination of agricultural land [5-7]. When crops uptake Cd, this can lead to high levels of Cd in the human food chain [7]. In Japan, itai-itai disease was caused by Cd contamination, and resulted in the deaths of several hundred people in the 1950s [8]. Therefore, the contamination of the food chain with Cd is considered a major threat to human health [5].

In recent years, heavy metal-resistant bacteria and silicon (Si) have become known as biologically safe tools to mitigate heavy metal toxicity in plants [10, 11]. The application of Si has significant beneficial effects on several plant species. It has been reported that Si reduces the detrimental effects of various heavy metals like manganese [12], aluminium [13], zinc [14], and copper [15]. There are several reports about the key role of Si in alleviating Cd toxicity in plants through a reduction in uptake of cadmium [16-18]. Si plays a key role in alleviating Cd toxicity by also decreasing Cd accumulation in the plant shoot, and by stimulating the plant’s defense system [19]. The Earth’s crust is rich in silicate and phosphate. However, plants can only absorb Si as monosilicic acid (H_4_SiO_4_) [20] and phosphorus (P) as H_2_PO_4_ and HPO_4_^2-^ [21]. On the other hand, the massive use of P fertilizer causes global economic and environmental problems such as increased P prices, higher agricultural production costs and elevated net accumulation of P in soil and heavy metal toxicity [22, 23]. Moreover, significant loss of P through leaching and runoff from agricultural land generates huge discharges into water bodies that accelerates eutrophication [24]. An overview of these scenarios in terms of Si and P fertilizer application represents lower utilization of resources, extravagant investment and threat to the environment and food chain. Therefore, there is undoubtely an urgent need for fertilizer that can eliminate the metal toxicities from crops and enhance productivity in an eco-friendly manner along with the amelioration of the soil toxicity problem.

Plant growth promoting rhizobacteria (PGPR) have recently been considered as eco-friendly bioferilizer that tends to immobilize heavy metals and inhibit its translocation in plants [25]. Moreover, PGPR upregulate the plant defense system through stimulation of antioxidant activity, modulation of phytohormones and regulation of metal transporter genes [1, 26]. PGPR are reported to confer resistance to plants under heavy metal stress through production of plant hormones such as indole-3-acetic acid (IAA) and gibberellins. In addition, PGPR produce organic acid, 1-aminocyclopropane-1-carboxylate (ACC)-deaminase, and solubilize phosphate that enhance defense mechanisms and minimize heavy metal translocation within the plant tissues [1, 27]. Among PGPR, phosphate solubilizing bacteria have been widely reported to confer tolerance in plants under biotic and abiotic stress [28]. However, studies on silicate solubilizing bacteria are largely ignored. Since researchers of the past few decades have highly prioritized the importance of silicon especially in a high silicon-accumulator crops like rice, their studies have focused on sustaining the silicon and phosphorus demand in plants through the application of silicate and phosphate solubilizing PGPR in a rice plants.

Rice is the most stable cereal crop plant in the world, feeding approximately half of the global population [29]. Exposure of Cd in environment had led to toxic rice production, which upon intake may impose a severe threat to humankind. [30]. In addition to toxic production, Cd also alters the normal physiology of plants. In general, when Cd is accumulated by plants it causes phytotoxicity, lowers plant nutrient uptake, and inhibits the photosynthetic process [31, 32]. Several studies have showed that metal resistant bacteria that are involved in phytohormone production and phosphate solubilization stimulate plant growth and mitigate heavy metal toxicity [33, 34]. In the current study, we have selected the strain that could resist heavy metal and has high ability for silicate and phosphate solubilization, as well as IAA and gibberellin (GA) production [35].

Here, we demonstrate how silicate and phosphate solubilization in soil through microorganisms could minimize the Cd uptake and improve the physiology of rice plants under cadmium stress. Our work shows that the application of silicate and phosphate solubilizing bacteria could solubilize insoluble silicate and phosphate in soil, and thereby reduce the requirement for phosphate fertilizer application, mitigate Cd toxicity, and enhance the growth and production quality of the plants.

## Materials and Methods

### Silicate and Phosphate Solubility Performance Test

The bacterial strain *Enterobacter ludwigii* GAK2, which was registered with accession number KP 676113 in the NCBI database,(Lee 2015) https://www.ncbi.nlm.nih.gov/nuccore/830699107, was selected for conducting the experiment. The bacterial bioassay of resistance to heavy metals was perfomed in glucose media (glucose 10 g/l, with 0.25% Mg_2_O_8_Si_3_) and National Botanical Research Institute’s Phosphate (NBRIP) growth media [glucose 10 g/l, MgCl_2_·6H_2_O 5 g/l, MgSO_4_·7H_2_O 0.25 g/l, KCl 0.2 g/l,(NH_4_)_2_SO_4_ 0.1 g/l with 0.25% Ca_3_(PO_4_)_2_] with or without Cd with random concentrations from 100 µM to 1,500 µM. Paper discs were placed on media plates and 20 µl of each bacterial culture (optical density >1, at 660 nm measured by UV spectrophotometer, PG instrument T60U, UK) was added and incubated at 30°C. A visual assessment of solubilization ability through observing a clear zone in the media plates was performed and used for further testing.

### Experimental Design

The effect of GAK2 was investigated with insoluble silicate (IS) and insoluble phosphate (IP) along with cadmium. The experiment involved the following treatments: Control (Cd), IS+Cd (Mg_2_O_8_Si_3_ and Cadmium), GAK2+Cd (Bacteria and Cadmium), IS+GAK2+Cd (Mg_2_O_8_Si_3_ + Bacteria + Cd), IP + Cd (Ca_3_(PO_4_)_2_ and Cadmium), IP+GAK2+Cd (Ca_3_(PO_4_)_2_ + Bacteria+Cadmium).

### Determination of Soil pH for Plant Experiment

The selection of appropriate doses of Mg_2_O_8_Si_3_ or Ca_3_(PO_4_)_2_ was based on the previous study done by Lee *et al*. [29]. Here, autoclaved (121°C, 15 min, 3 times) nursery paddy soil (200 g per pot) was placed in sealed pots with a size of 10 × 10 cm. Mg_2_O_8_Si_3_ or Ca_3_(PO_4_)_2_ in quantities of 0.0 g, 0.2 g, 0.4 g, 0.6 g, 0.8 g, 1.0 g, 2.0 g, were added to the 10 × 10 cm pots and mixed thoroughly. Distilled water (100 ml) was poured on each pot and the pH was measured after 24 h according to the procedure described by Kalra [36].

### Plant Material and Pot Experiment 

The Korean rice cultivar Hwayeongbyeo was selected for the pot experiment to investigate the effect of *E. ludwigii* GAK2 under cadmium stress. The rice variety was developed by the Rural Development Administration, South Korea. Yeongnam Agricultural Experiment Station, South Korea, reported the variety as a first generation of cultivar Chukei 830 and YR 4811 Acp 8. The autoclaved soil (121°C, 15 min, 3 times) and similar pots as mentioned in section 2.3 were filled with 200 g autoclaved (121°C, 15 min, 3 times) nursery paddy soil for the current experiment. To each pot, 0.4 g of either Mg_3_Si_2_O_8_ or Ca_3_(PO_4_)_2_ was added and two-week-old rice seedlings were transplanted.

Bacterial culture (grown on LB of O.D > 1) was kept in a 250 ml bottle, and centrifuged at 6,000 ×g for 10 min at 4°C. The pellets obtained were diluted with sterilized with distilled water to form 108 colony-forming units (CFU) per milliliter. Five days after seedling transplantation, 50 ml of freshly diluted bacterial culture was inoculated to each pot; this was repeated after a further 5 days. Five days after the second inoculation, 80 ml of 1 mM CdSO_4_·8H_2_O was added to each pot for 7 consecutive days. After 5 days the plants were harvested, and their morphological parameters (root length, shoot length, fresh root and shoot weight, plant height) and chlorophyll content (SPAD-502, Konica Minolta, Japan) were measured. Fresh samples were separated for RNA extraction and others were lyophilized, ground, and further biochemical analysis was performed. The entire experiment was conducted in a growth chamber (KGC-175 VH, KOENCON) and conditions were programmed for a 12-h light (08:00~20:00; 30°C; relative humidity 68%), 12-h dark (20:00~08:00, 24°C; relative humidity 68%) cycle.

### Quantification of Cadmium Content in Rice Plants 

The method described by Kang *et al*. [37] was followed to quantify the Cd content. Lyophilized and powdered rice samples (0.5 g) were soaked briefly with 0.5 M HCl, and rinsed through double distilled water before oven drying. A mixture of nitric acid, sulfuric acid and perchloric acid at (10:1:4 v/v/v) was subjected through the sample. The digested sample obtained was then analyzed with an Inductively Coupled Plasma machine (Optima 7900DV, Perkin-Elmer, USA).

### RNA Isolation and qPCR Analysis of Selected Genes 

The RNA was extracted by following the protocol described by Chan *et al*. [38] . The expression level of Si-carrying gene (*OsLsi1*), Cd-carrying gene (*OsHMA2*), and jasmonic acid related gene (*OsJAZ1*) were analyzed by qPCR [39]. In brief, cDNA was synthesized using a qPCRBIO cDNA Synthesis Kit following the manufacturer’s instructions. qPCRBIO SYBR Green Kit from PCRBIOSYSTEM was used following the manufacturer’s instructions to conduct the reaction. q-PCR was conducted using Illumina Eco Real-Time PCR System (Singapore), to relatively quantify the expression level of selected genes. The primers of *OsLsi1* (Accession No. N17-020055), *OsHMA2* (Accession No. XM-015788173) and *OsJAZ1* (Accession No. XM-015757562) were used as a reference (Table S2). To standardize the level of expression, *OsActin* was used as housekeeping gene. The total volume of the reaction was 20 µl containing 7 µl ddH_2_O, 1 µl primer, 10 µl SYBR Green and 1 µl cDNA.

### Jasmonic Acid (JA) Quantification 

Standard protocol of McCloud and Baldwin [40] as described in Shahzad *et al*. [41] was followed to quantify the JA content in the plants. Briefly, 0.1 g of freeze-dried sample was extracted with acetone, followed by the addition of dd.H_2_O and JA standard. The acetone was evaporated, and 0.1 M phosphate buffer was added, and the pH was adjusted to between 2 and 3. Chlorophyll was removed with diethylaminoethyl (DEAE) cellulose and partitioned with chloroform. The solution was then run through an NH2 cartridge and finally quantified with a gas chromatography select ion monitoring (GC-SIM) (6890N network GC system and the 5973 network mass selective detector; Agilent Technologies, USA).

### Statistical Analysis

The present study was conducted in a completely randomized design in which each treatment had 8 replicates. Statistical analysis was performed with the program R and the data are presented as the means ± standard deviation (SD). Significant differences among treatments were determined using the least significant difference (LSD) method at *p* ≤ 0.05.

## Results

### Bioassay of *Enterobacter ludwigii* GAK2

**Fig. 1:**
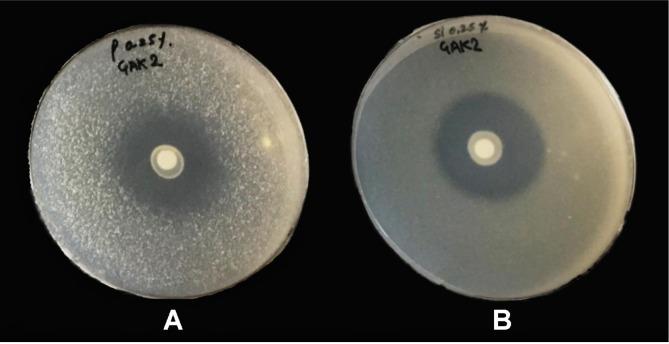
Screening of silicate and phosphate solubility performance test of *Enterobacter ludwigii* GAK2 under Cd stress 1,000 µM. (**A**) Silicate solubilization test on glucose agar media plate with 0.25%magnesium trisilicate. (**B**) Phosphate solubilization test on NBRIP agar media plate with 0.25% calcium phosphate (clear zone on the media plate represents the solubilization ability of *Enterobacter ludwigii* GAK2).

Our research revealed that *E. ludwigii* GAK2 could solubilize the insoluble silicate and phosphate in the presence of cadmium (1 mM CdSO_4_·8H_2_O). Based on these results, 1 mM CdSO_4_·8H_2_O was applied during the plant experiment. The clear, circular zones formed around the bacterial colonies indicated silicate solubilization in the glucose media with silicate, and phosphate solubilization in the NBRIP media with phosphate, respectively (Fig. 1).

### Determination of pH Value at Different Concentrations of Ca_3_(PO_4_)_2_ and (Mg_2_O_8_Si_3_)

**Fig. 2:**
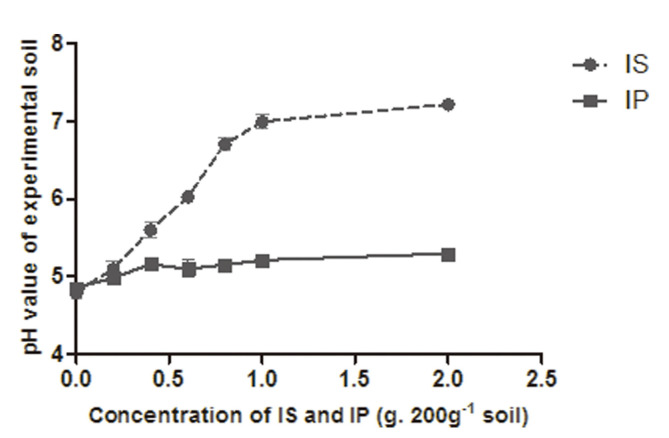
pH value of soil under different concentrations of insoluble phosphate (IP) and insoluble silicate (IS).

The pH value of soil increased as the amount of added Mg_2_O_8_Si_3_ increased. The application of 0.4 g of Mg_2_O_8_Si_3_ resulted in a soil pH between 5.5-6. However, the application of Ca_3_(PO_4_)_2_ did not influence the pH value of soil (Fig. 2).

### Analysis of Cadmium Content in Rice Shoots

**Fig. 3:**
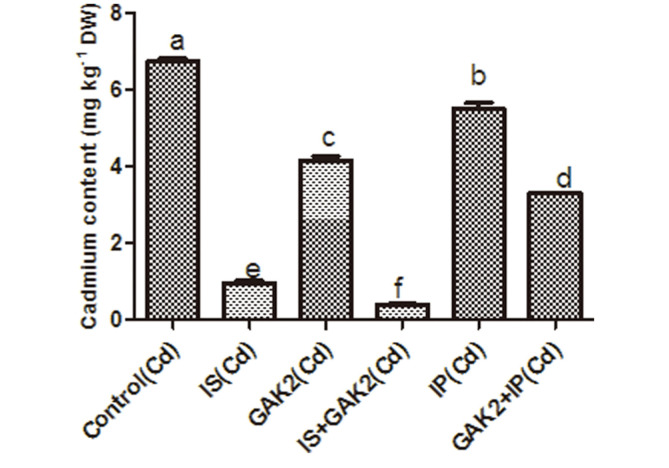
Quantification of cadmium content of rice shoot.

This study showed that Cd content was significantly higher in control plants, but upon inoculation of GAK2, the Cd content was significantly reduced by 34%. Approximately 94% Cd uptake was decreased in plant shoots grown on the IS amended soil with bacterial inoculation (IS + GAK2), as compared to control. Likewise, Cd content was significantly reduced by 51% in plant shoots grown on the IP amended soil with bacterial inoculation (IP + GAK2) (Fig. 3). Compared to the control, the sole application of IS and IP reduced the cadmium content by 86% and 18% respectively.

### Silicon and Cadmium Carrying Gene Expression Analysis

**Fig. 4:**
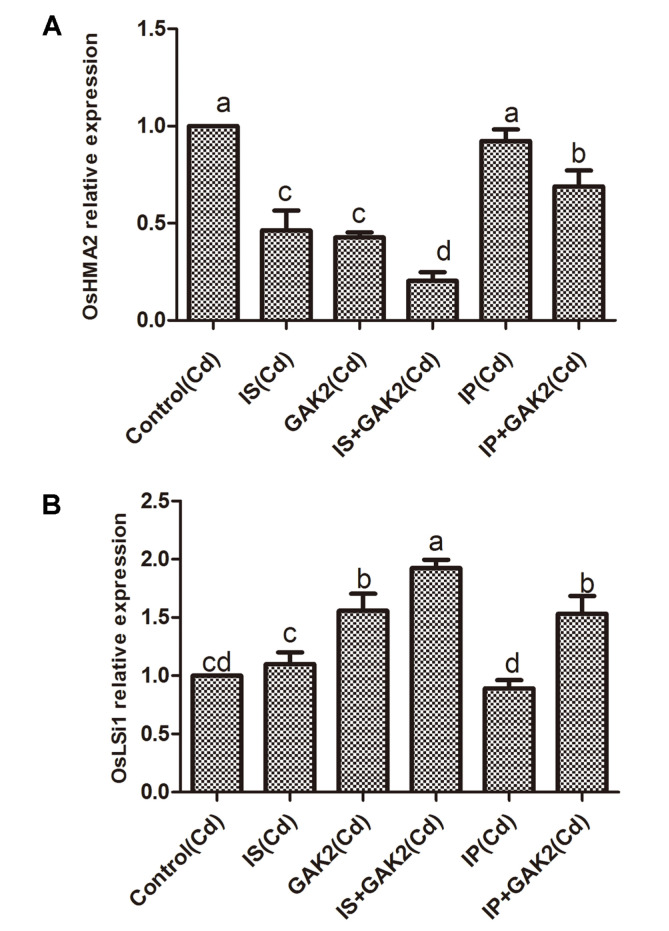
Expression of cadmium transporter gene (**A**) *OsHMA2* and (**B**) silicon-carrying *OsLsi1* gene in rice plants (*Oryza sativa*).

The expression of the silicon transporter gene, *OsLsi1* was found to be significantly higher in IS + GAK2 and in IP+GAK2 compared to that in the control. The Cd transporter gene, *OsHMA2* showed significantly lower expression in IS+GAK2 and IP + GAK2 when compared to control. Moreover, the sole application of IS significantly reduced the expression of *OsHMA2*; however, no significant difference was observed in *OsLsi1* expression (Fig. 4).

### Plant Growth Promoting Attributes

The plant growth promoting attributes of fresh root weight, fresh shoot weight, plant height, and chlorophyll content were all significantly reduced in the Cd stressed plants. However, all these attributes were significantly increased when treated with GAK2. Under Cd stress, IS+GAK2 significantly improved plant growth (fresh root weight 197%, fresh shoot weight 61%, height 14%, and chlorophyll content 23%) compared to those of the control. Similar trends were observed in GAK2+IP application which significantly increased (fresh root weight 147%, fresh shoot weight 67%, plant height 7.6%, and chlorophyll content 34%) when compared with control. The sole application of GAK2, IS and IP also promoted the plant growth promoting characteristics under Cd stress. Overall, combined application of IS and GAK2 has significant effect on plant growth among all the treatments (Table 1 ).

### Jasmonic Acid (JA) Analysis 

**Fig. 5:**
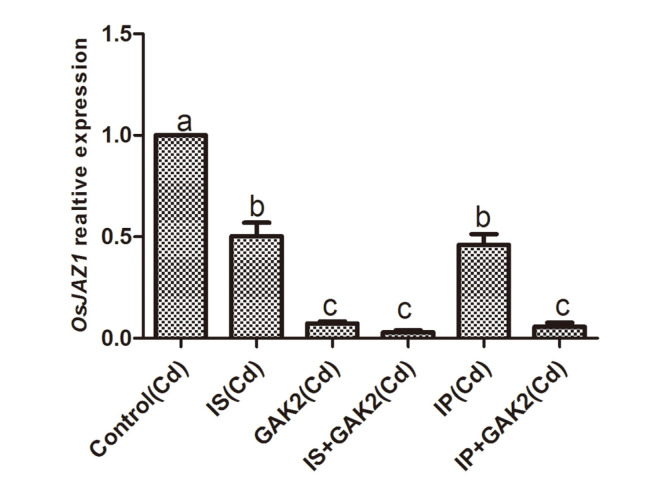
Jasmonic acid related *OsJAZ1* gene expression of rice shoot.

**Fig. 6:**
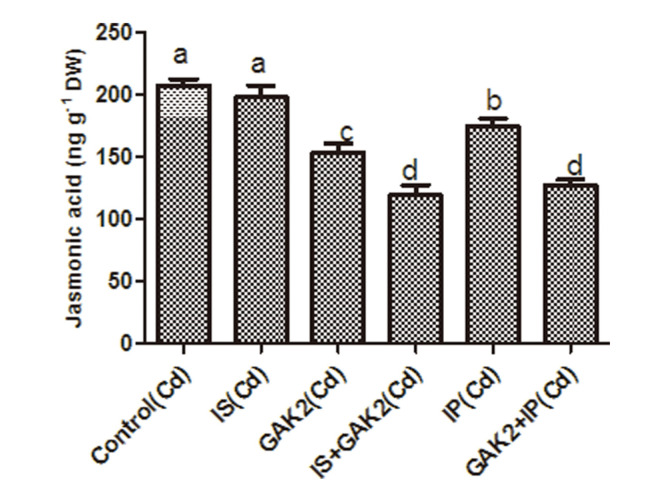
Quantification of jasmonic acid of rice shoot.

Under Cd stress, the JA content of the Cd stressed plants was significantly higher when compared to control. However, inoculation of GAK2 solely and along with IS and IP significantly reduced the JA content when compared to control. No significant difference was observed on sole application of IS. Sole application of GAK2 reduced JA content by 26%. Similarly, JA content was significantly reduced by 39% in IP+GAK2 and by 42% in IS+GAK2. Furthermore, the levels of JA were correlated with the JA related *OsJAZ1* expression. Expression of *OsJAZ1* was significantly higher in the Cd stressed plants. However, the expression of *OsJAZ1* was significantly reduced in IS+GAK2 and IP+ GAK2 when compared to control (Figs. 5 and 6).

## Discussion

Si is considered an essential nutrient for plants, especially in rice, which is also known as a Si hyperaccumulator [42]. It has been reported that only 10-30% of phosphate fertilizer is utilized by plants, whereas the remaining fertilizer is rapidly converted into a form which is unavailable for plant uptake [43, 44]. We attempted to meet the Si and P demands of rice through the solubilization of insoluble silicate and phosphate. Through screening, it was found that the strain *E. ludwigii* GAK2 could efficiently solubilize silicate and phosphate, in glucose and NBRIP media. A similar method of solubilization process was reported by Naureen *et al*. [45], who found that acid producing bacteria formed a clear zone through silicate solubilization. Moreover, the strain GAK2 possesses an innate ability to survive in the presence of heavy metals including Cd, Ni, Zn, and Cu (Figs. S1 and S2). We also found that GAK2’s solubilization ability decreases as the heavy metal concentration increases. Nevertheless, GAK2 could efficiently solubilize silicate and phosphate, up to concentrations of 1,000 µM CdSO_4_·8H_2_O, on glucose and NBRIP media, respectively. Therefore, GAK2 was further investigated by inoculation in ‘Hwayeongbyeo’ rice plants to test mitigation effects against Cd stress on IS and IP mediated soil.

The issue of heavy metal toxicity is of global concern. The presence of Cd ions is responsible for interfering with the uptake, transport, and distribution of several mineral nutrition elements in plants [46, 47] . In the current study, the suppression of Cd uptake in rice shoots because of the application of GAK2, with either IS or IP, indicates that GAK2 plays a key role in mitigating phytotoxicity in plants. There are several reports that have mentioned the beneficial effect of Si on the reduction of heavy metal concentration in plants. Since GAK2 has the ability to solubilize the insoluble silicate to soluble form, the possible Si-plant interaction might have lowered the Cd content in the plant. The *OsLsi1* gene is reportedly used for Si transmission, and *OsHMA2* for Cd transport in rice [39]. In this study, the higher expression of *OsLsi1*, and lower expression of *OsHMA2*, on inoculation with GAK2, is further evidence of silicate and phosphate dissolution. Moreover, organic compounds are reported to play a key role in metal solubilization [48]. It has been already reported that *Enterobacter ludwigii* GAK2 has the ability to produce organic acids, like acetic acid, citric acid, and lactic acid [49]. These acids might have played a role in metal regulation in plants.

Metal-resistant microorganisms have various strategies to colonize heavy metal-polluted soils according to Meharg and Cairney [50]. Heavy metal-resistant strains also help plants to gain nutrients and phytohormones for optimal growth as reported by Zaidi *et al*. [51]. In our study, attributes associated with plant growth like root and shoot weight, plant height, and chlorophyll content were found to be significantly higher on the GAK2 treated plants with IS or IP. Our results are in line with previous findings by Madhaiyan *et al*. [52], where *Burkholderia* sp. strain CBMB40 and *Methylobacterium oryzae* strain CBMB20 mitigated Cd and Ni toxicity, and promoted growth in rice. In our previous study, it was found that *E. ludwigii* GAK2 could produce IAA and biologically active gibberellin(GA) [35]. Since, IAA and GA are widely reported to enhance plant growth, synthesis of GA and IAA by *E. ludwigii* GAK2 on the plant root rhizosphere could be one possible reason to confer tolerance and to stimulate growth. Moreover, our results are in agreement with Kang *et al*. [37], who showed that the silicate solubilizing bacteria *Burkholderia eburnea* CS4-2 promoted rice plant growth through silicate solubilization.

The presence of heavy metal usually causes stress to plants, inducing several metabolic changes as reported by Madhaiyan *et al*. [52]. During plant-microbe interaction, JA plays an important role in mitigating biotic and abiotic stresses. In the present study, GAK2 inoculation significantly lowered the JA content on IS and IP mediated soil. Similarly, JA related gene *OsJAZ1* expression was lowered by IS + GAK2 and IP + GAK2 treatment. Shahzad *et al*. [41] reported that the gibberellin producing *Bacillus amyloliquefaciens* modulates the phytohormones, and lowered the JA content, in plants. Since, GAK2 has been reported for functional gibberellin production these might have played a possible role in JA regulation in plants. Moreover, similar trends were reported by Kang *et al*. [53], who found that endogenous JA content was lowered by the inoculation of gibberellin producing microbes.

Soil pH is considered as one of the most important factors affecting the availability of Cd [54]. It was reported that at pH 7.0 the metal removal rate of Cu, Pb, Zn, and As were high [55]. Numerous investigations have reported that there is an inverse relationship between Cd and soil pH, as an increase in soil pH decreases the Cd concentration in plants. However, it has also been reported that although the availability of Cd in soil is affected by pH, the increase in pH level of soil does not always reduce metal uptake by plants [54]. The sorption of Cd is affected by ionic strength, which varies widely among different soils [56]. Since silicate contributes to an increase in the soil pH level, this reduces the level of heavy metal uptake by the plants. However, accumulation depends on the genotype of a plant [57].

The abundance of silicate in the Earth’s crust, and heavy metal pollution caused by phosphate fertilizer application, are both well-established facts. This study revealed that the *Enterobacter ludwigii* GAK2 could effectively solubilize the silicate and phosphate in soil, and thereby promote the growth of plants in Cd contaminated soil. In conclusion, this bacterial strain could be a suitable silicate and phosphate bio-fertilizer, that may reduce the requirement for the application of synthetic fertilizers, and decrease the Cd concentration in soil and crops. An extensive study is suggested on the spatial and temporal aspects of *Enterobacter ludwigii* GAK2, along with various forms of silicate, phosphate, and Cd in association with different crop plants.

## Supplemental Materials



Supplementary data for this paper are available on-line only at http://jmb.or.kr.

## Figures and Tables

**Table 1:** Effect of *Enterobacter ludwigii* GAK2 on plant growth promoting attributes in rice plants (*Oryza sativa*).

Treatment	F. Sh. Wt. (g/plant)	PH (cm/plant)	Chl.(SPAD)	F.Rt.Wt. (g/plant)
Control(Cd)	2.83 ± 0.31^c^	52 ± 2^c^	34 ± 2.65^c^	0.4 ± 0.1^d^
IS(Cd)	3.85 ± 0.28^b^	57.33 ± 2^a^	37.7 ± 4.3^bc^	0.67 ± 0.06^cd^
GAK2(Cd)	3.80 ± 0.36^b^	56.33 ± 2.08^ab^	41.13 ± 1.5^ab^	0.8 ± 0.09^bc^
IS+GAK2 (Cd)	4.53 ± 0.31^a^	59.33 ± 1.53^a^	41.93 ± 3.44^ab^	1.19 ± 0.25^a^
IP(Cd)	4.2 ± 0.45^ab^	53 ± 3^bc^	37.9 ± 1.13^bc^	0.77 ± 0.10^bc^
IP+GAK2(Cd)	4.73 ± 0.35^a^	56 ± 2^ab^	45.53 ± 4.01^a^	0.98 ± 0.11^ab^

F.Sh.Wt: Fresh Shoot Weight, PH: Plant Height, Chl: Chlorophyll Content, F.Rt.Wt: Fresh Root Weight, GAK2: Bacteria, IS: Insoluble silicate (Magnesium trisilicate), IP: Insoluble Phosphate (Calcium phosphate), Cd: Cadmium, SPAD: Soil Plant Analysis Development Chlorophyll Meter. The mean values followed by different superscripts in the same column represent significant differences (*p* ≤ 0.05). Each value represents mean ± SD (*n* = 6).
