# Supplementary material for: Effect of Silicate and Phosphate Solubilizing Rhizobacterium Enterobacter ludwigii GAK2 on Oryza sativa L. under Cadmium Stress
Source: J Microbiol Biotechnol. 2019 Oct 25;30(1):118–26. doi: 10.4014/jmb.1906.06010 (PMC9728408; doi:10.4014/jmb.1906.06010)
Supplement: Supplementary file 1 [file JMB-30-1-118-supple.pdf]

J. Microbiol. Biotechnol.
https://doi.org/10.4014/jmb.1906.06010

jmb

Supplementary information: We would like to submit the details and data supplemental to the main text that would disrupt the flow of the main text, but nonetheless remain crucial to understanding.

Firstly, we screened the *E. ludwigii* GAK2 ability to solubilize silicate and phosphate under various heavy metal like Zn, Cu, Ni and Cd with different concentration. Based on these screening results we have selected the cadmium concentration 1000 µM for our plant experiment. One another reason for selecting 1000 µM Cd concentration is due to the low diameter of clear zone formed by GAK2 strain above 1000 µM Cd concentration within same period of time. **Supplementary Fig. (1, 2).** The silicate and phosphate solubilizing index was determined under 1000 µM Cd stress according to the method described by Pande et al. [1] (**Supplementary Table 1)**. In addition, in our previous study Lee et al. [2], reported the GAK2 strain ability to produce IAA, we further confirmed the IAA production test under different Cd concentration 500µm,1000µm, 1500 µm through salkowski reagent test. The visual assessment in change in the red color of GAK2 culture broth represent the strain ability to produce IAA under cadmium stress (**Supplementary Fig. 3)**. In addition, we have further identified the *E. ludwigii* potentiality for 1-aminocyclopropane -1-carboxylate (ACC) deaminase production (**Supplementary Fig. 4).** The method described by Li et al. [3] was followed for the detection of ACC deaminase activity. *acdS* genes *acdS*f3, 5′-ATCGGCGGCATCCAGWSNAAYCANAC-3′ and *acdSr3*, 5′-GTGCATCGACTTGCCCTCRTANACNGGRT-3′ was used for gene amplification. Moreover, we also detected the Abscisic acid(ABA) level on plant shoot. The method described by Shahzad

et al. [4] was followed to extract and quantify the ABA. The results showed that *E. ludwigii* GAK2 inoculation along with IS or IP significantly reduced the ABA level on the plant shoot (**Supplementary Fig. 5)**. We believe that these results would put more insight on the understanding of our research. Based on these facts further experiment would be carried out on the near future.

1. Pande A, Pandey P, Mehra S, Singh M, Kaushik S. 2017. Phenotypic and genotypic characterization of phosphate solubilizing bacteria and their efficiency on the growth of maize. *Journal of Genetic Engineering and Biotechnology*. **15:** 379-391.
2. Lee K-E, Adhikari A, Kang S-M, You Y-H, Joo G-J, Kim J-H, *et al.* 2019. Isolation and Characterization of the High Silicate and Phosphate Solubilizing Novel Strain Enterobacter ludwigii GAK2 that Promotes Growth in Rice Plants. *Agronomy*. **9:** 144.
3. Li Z, Chang S, Ye S, Chen M, Lin L, Li Y, *et al.* 2015. Differentiation of 1-aminocyclopropane-1-carboxylate (ACC) deaminase from its homologs is the key for identifying bacteria containing ACC deaminase. *FEMS microbiology ecology*. **91:** fiv112.
4. Shahzad R, Khan AL, Bilal S, Waqas M, Kang S-M, Lee I-J. 2017. Inoculation of abscisic acid-producing endophytic bacteria enhances salinity stress tolerance in Oryza sativa. *Environmental and Experimental Botany*. **136:** 68-77.

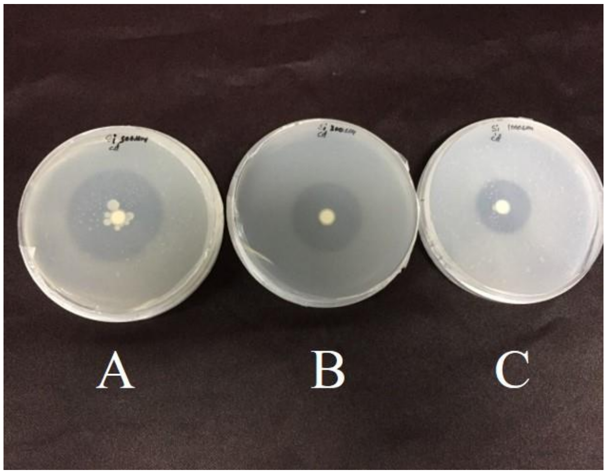

Supplementary Fig. 1. Silicate solubilization test under Cd stress (A) 200 µM, (B) 500 µM, (C) 1000 µM. (Clear Zone on the media plate represents the silicate solubilization area by *Enterobacter ludwigii* GAK2).

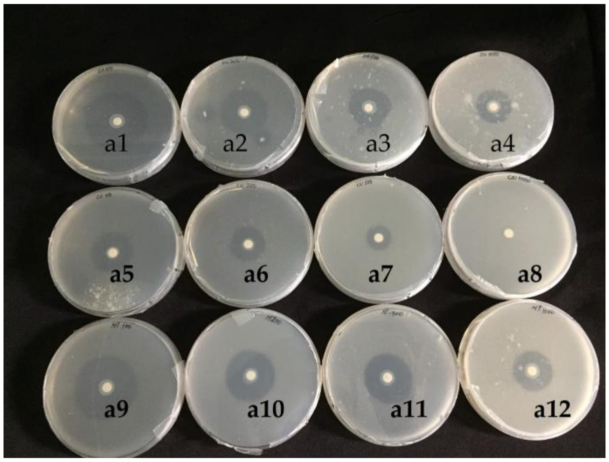

Supplementary Fig. 2. Silicate solubilization test under Zinc [(a1) 200 µM, (a2) 500 µM, (a3) 1000 µM, (a4) 1500 µM], Copper [(a5) 200 µM, (a6) 500 µM, (a7) 1000 µM, (a8) 1500 µM], and Nickel[(a9) 200 µM, (a10) 500 µM, (a11) 1000 µM, (a12) 1500 µM] amended glucose agar media plate containing 0.25% magnesium trisilicate. (Clear Zone on the media plate represents the silicate solubilization area by *Enterobacter ludwigii* GAK2).

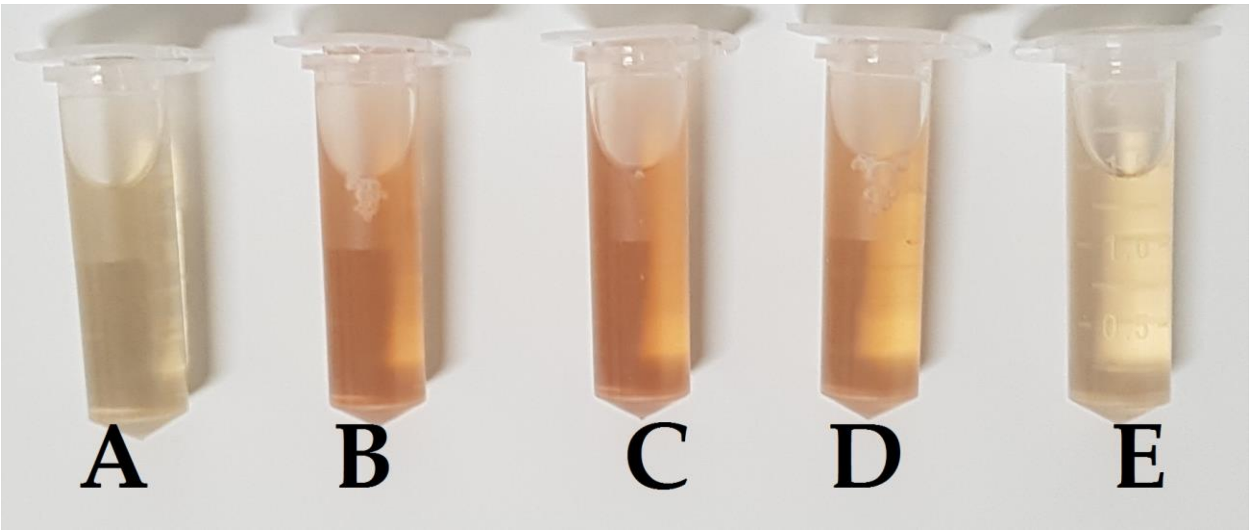

Supplementary Figure 3. Visual assessment of Indole-3-acetic acid production of *Enterobacter ludwigii* GAK2 culture media through salkowski reagent test under diffrent $CdSO_4.8H_20$ concentration (A) Non-inoculated media (B) Control (C) 500 µM Cd stress (D) 1000 µM Cd stress (E) 1500 µM Cd stress.

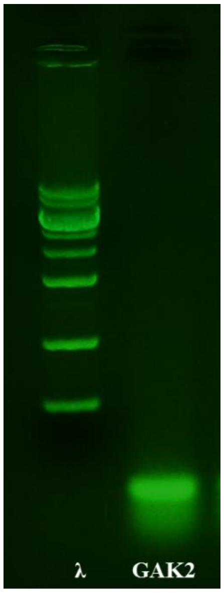

Supplementary Figure 4. Detection of ACC deaminase activity through amplification of *acdS* genes(*acdS*f3, 5′-ATCGGCGGCATCCAGWSNAAYCANAC-3′ and *acdSr3*, 5′-GTGCATCGACTTGCCCTCRTANACNGGRT-3′).

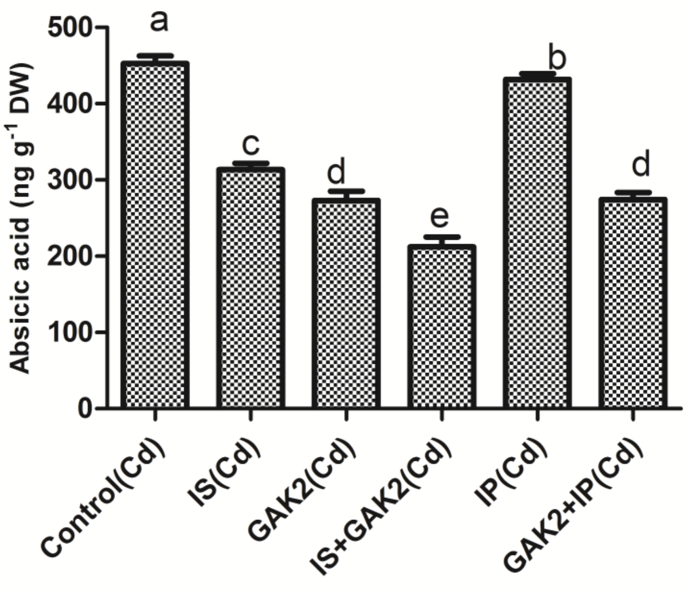

Cd: Cadmium**, GAK2**: Bacteria, **IS**: Insoluble silicate (Magnesium trisilicate), IP: Insoluble Phosphate (Calcium phosphate)

Supplementary Figure 5. Quantification of abscisic acid of rice shoot. Different letters above the bar diagram represent significant differences at $p < 0.05$. Bars represent mean±SD (n = 6).

Supplementary Table 1. Measurement of Phosphate and Silicate solubilizing index of *E. ludwigii* GAK2 under 1000 µM Cd stress

**Bacterial culture media**	**Colony diameter(cm)**	**Clear zone diameter (cm)**	**Phosphate solubilization index**	**Silicate solubilizing index**
NBRIP media	0.49±0.09	1.8±0.10	4.6	
Glucose media	0.52±0.04	2.06±0.15		4.9

Each measurement were recorded in triplicates.

Supplementary Table 2. Details of the primer used in current experiment

**Name**	**Accession**	**Forward Primer (5´-3´)**	**Reverse Primer (5´-3´)**
OsHMA2	XM-015788173	CATAGTGAAGCTGCCTGAGATC	GATCAAACGCATAGCAGCATCG
OsJAZ1	XM-015757562	CCCGGAGATGCCGAT	CATACTATGCATAGAAATGGAGAC
OsLsi1	N17-020055	CGGTGGATGTGATCGGAACCA	CGTCGAACTTGTTGCTCGCCA
